# Modulators of Transient Receptor Potential (TRP) Channels as Therapeutic Options in Lung Disease

**DOI:** 10.3390/ph12010023

**Published:** 2019-02-01

**Authors:** Alexander Dietrich

**Affiliations:** Walther-Straub-Institute of Pharmacology and Toxicology, Member of the German Center for Lung Research (DZL), LMU Munich, Nussbaumstr. 26, D-80336 Munich, Germany; Alexander.Dietrich@lrz.uni-muenchen.de; Tel.: +49-89-2180-73802

**Keywords:** non-neuronal, transient receptor potential (TRP) channels, TRPA1, TRPC6, TRPV1, TRPV4, lung, asthma, cystic fibrosis, lung fibrosis, lung edema, chronic obstructive pulmonary disease (COPD)

## Abstract

The lungs are essential for gas exchange and serve as the gateways of our body to the external environment. They are easily accessible for drugs from both sides, the airways and the vasculature. Recent literature provides evidence for a role of Transient Receptor Potential (TRP) channels as chemosensors and essential members of signal transduction cascades in stress-induced cellular responses. This review will focus on TRP channels (TRPA1, TRPC6, TRPV1, and TRPV4), predominantly expressed in non-neuronal lung tissues and their involvement in pathways associated with diseases like asthma, cystic fibrosis, chronic obstructive pulmonary disease (COPD), lung fibrosis, and edema formation. Recently identified specific modulators of these channels and their potential as new therapeutic options as well as strategies for a causal treatment based on the mechanistic understanding of molecular events will also be evaluated.

## 1. Introduction

**Figure 1:**
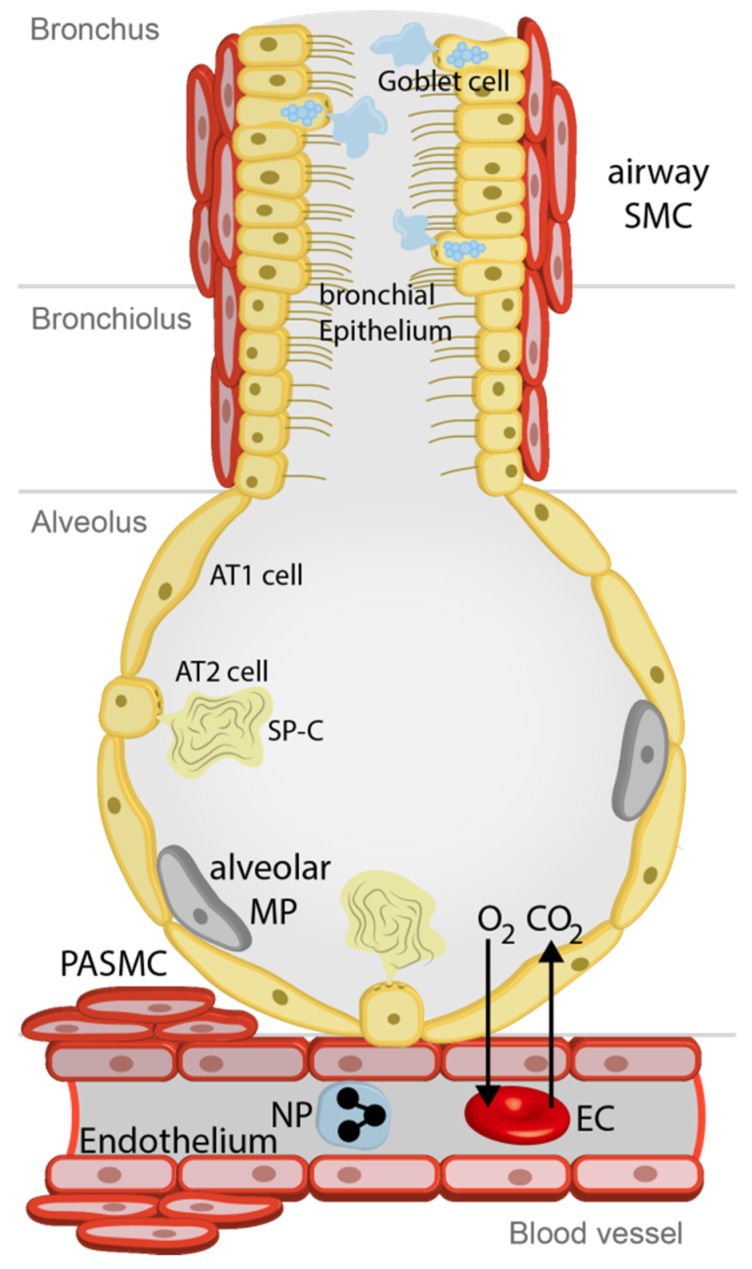
Cells involved in physiological functions of airways and lungs. See text for more details. Airway SMC, airway smooth muscle cells; Alveolar MP, alveolar macrophages; AT1 cells, alveolar type 1 cells; AT2 cells, alveolar type 2 cells; CO_2_, carbon dioxide; EC, erythro-cyte; O_2_, oxygen; NP, neutrophil; PASMC, precapillary arterial smooth muscle cells, SP-C, surfactant protein-C.

Our lungs are not only essential for gas exchange but are also exposed to the external environment. Therefore, we inhale toxicants, allergens, and infectious agents like bacteria, viruses, and fungi together with vitally important oxygen. The airways of our lungs are composed of bronchi, which branch many times into smaller airways ending in narrow bronchioles. At the end of each bronchiole are thousands of small air sacs, called alveoli, where the gas exchange with the circulating blood takes place. In the upper airways, ciliated epithelium together with mucus producing goblet cells try to extrude foreign particles supported by the cough reflex, which is triggered by activation of sensory nerve endings [1]. In the alveoli, thin aveolar type 1 (AT1) epithelial cells ensure efficient gas exchange, while square-sized alveolar epithelial cells type 2 (AT2) produce surfactant protein to reduce surface tension and to serve as an additional barrier. Moreover, AT2 cells are able to differentiate to AT1 cells to rebuild the alveolar barrier after acute or chronic injury [2]. Next to the airways, the pulmonary vasculature ensures fast transport of oxygen by erythrocytes to organs in the body replacing CO_2_, which is exhaled (see Figure 1). Airway epithelium and vascular endothelium offer a most efficient protection of the lung, which will be seriously damaged if cellular functions are clogged. In all these cells and additionally in immune cells circulating in the pulmonary vasculature, members of the superfamily of transient receptor potential (TRP) channels are expressed. These non-selective Ca^2+^ permeable cation channels at the plasma membrane have multiple functions as chemosensors and essential members of signal transduction cascades triggering cell function in response to stimuli. They are, however, also involved in pathophysiological processes of the lung (like chronic inflammation, fibrosis, and edema). This review article, which is also an update of our former manuscript [3], will focus on TRP channels (TRPA1, TRPC6, TRPV1, and TRPV4) predominantly expressed in non-neuronal lung tissues. I will also highlight their specific modulators, which may be important therapeutic options for lung diseases like pulmonary hypertension (PH), asthma, cystic fibrosis (CF), chronic obstructive pulmonary disease (COPD), lung fibrosis, and edema formation in the future. For other TRP channels mainly expressed in neuronal tissues of the airways and their involvement in the cough reflex, please refer two other recent reviews [4,5,6]. TRP channels as toxicant sensors in the lung are also highlighted in two other recent manuscripts [3,7].

## 2. TRP Expression in Lung Cells, Their Proposed Functions and Specific Regulators

TRP channels were first described in the fruit fly *Drosophila melanogaster* after characterization of a mutant with a short-lived depolarizing current as a visual defect termed transient receptor potential (TRP) [8]. This discovery led to the identification of Ca^2+^ permeable channels named TRP channels [9,10,11]. Ten years later in 1995, the first mammalian channel and founding member of the classical or canonical TRP family (TRPC1) was identified by homology screening in expressed sequence tag (EST) data bases (reviewed in [12]). Today, we know six TRP families with 28 different mammalian TRP channels. They are composed of intracellular N- and C-termini, six membrane-spanning helices (S1–S6), and a presumed pore forming loop (P) between S5 and S6 (reviewed in [13]). All TRPC family members harbor an invariant sequence, the TRP box (containing the amino acid sequence: EWKFAR), in its C-terminal tail as well as ankyrin repeats in the N-terminus [13]). For a functional TRPC ion channel complex, four monomers of the same type in a homotetrameric complex or four different TRPC monomers forming a heterotetrameric channel are essential [13]. All TRPC channels except TRPC1, whose role as ion channel or channel regulator is still a matter of debate [12], share a common activator diacylglycerol (DAG) [14,15] and are involved in complex cellular signal transduction cascades [16]. DAG is produced from phosphatidylinositol 4,5-bisphosphate (PIP_2_) by phospholipase C-isozymes activated after agonist binding to appropriate receptors [16].

The sixth member of the TRPC family, **TRPC6**, is highly expressed in lung tissues and its function was extensively studied in precapillary pulmonary arterial smooth muscle (PASMC) as well as in lung endothelial cells (LEC). PASMC essentially regulate blood pressure in the lung like small mesenteric arteries in the systemic vasculature. Therefore, we set out to isolate PASMC by transferring a well-established isolation procedure from rat [17] to mouse models. Most interestingly, PASMC predominantly express TRPC6 next to TRPC1 but only minor amounts of TRPC3 channels [18] in contrast to large pulmonary arteries. Therefore, a compensatory up-regulation of TRPC3 like in arteries of the systemic vasculature of TRPC6-/- mice did not arise and a true TRPC6-deficiency phenotype was identified. The Euler–Liljestrand reflex or acute hypoxic vasoconstriction of the pulmonary vasculature, which redirects blood flow from the hypoxic areas of the lung to normoxic regions was absent in these mice [18]. The reflex assures sufficient oxygen supply, if parts of the lungs are blocked by inhaled particles or by invading microorganisms, and if not functional induces lethal arterial hypoxemia in TRPC6-deficient mice [18]. In pulmonary endothelial cells, Ca^2+^ influx through TRPC6 increases cellular permeability induced by hypoxia. Pharmacological tools revealed a signal transduction cascade from hypoxia-induced Nox2-activation, production of reactive oxygen species (ROS), which induce PLCγ-phosphorylation and DAG-kinase-inhibition leading to DAG accumulation and TRPC6 channel-induced Ca^2+^-influx in endothelial cells of the lung [19]. Therefore, TRPC6 channels are indirectly activated by hypoxia and channel-induced Ca^2+^-influx is responsible for smooth muscle cell contraction and increases in endothelial permeability. 

While TRPC6 is only marginally expressed in fibroblasts, the channel is upregulated in myofibroblasts [20]. Initial results in cardiac and skin myofibroblasts [20] were reproduced in pulmonary myofibroblasts by my research group [21]. Next to these cells, TRPC6 is also expressed in alveolar macrophages [22], which are responsible for removing particles and microorganisms from the alveoli as well as in neutrophils [23,24], which migrate in lung tissues to fight against invading bacteria and viruses. Migration of TRPC6-/- neutrophils in response to macrophage inflammatory protein-2 (MIP2 also known as CXCL2) and CXR2-mediated chemotaxis was reduced in comparison to WT cells, while N-formyl-methionine-leucine-phenylalanine (fMLP) receptor-mediated chemotaxis was unchanged [23,24]. In summary, TRPC6 channels are highly expressed in lung tissues and are an interesting target for the development of therapeutic options in lung disease. Specific regulators of TRPC6 channels [25] are summarized in Table 1. Flufenamic acid is a rather unspecific TRPC/M/V channel blocker, but selectively activates TRPC6 and TRPA1 [25] and may be useful for the identification of channel activities in freshly isolated lung cells. Aniline-thiazoles [26] as well as small molecules from Glaxo-Smith-Kline (GSK2332255B and GSK2833503A) [27] block both closely related TRPC3 and TRPC6 channels. The latter compounds were already effective in reducing cardiac hypertrophy [27]. Another compound from Sanofi called SAR7334 effectively inhibited TRPC6 as well as TRPC3 and TRPC7 but not TRPC4 and TRPC5 [28]. More selective blockers are larixyl-derivates [29] especially larixyl N-methyl-carbamate with a nanomolar affinity to TRPC6 and a 13-fold subtype selectivity over TRPC3 [30]. Recently a small molecule inhibitor BTDM effective against TRPC3 and TRPC6 was identified, which binds to the S5–S6 pore domain of the human TRPC6 homotetramer analysed by single-molecule cryo-electron microscopy [31]. 

In contrast to TRPC6, expression of **TRPA1**, the only member of the TRPA family of TRP channels with a characteristic high number (16 in humans) of N-terminal ankyrin, repeats in lung is still elusive. Ankyrin repeats that are responsible for the letter A in the channel’s name are 33-amino-acid-motifs, which are found in many proteins and mediate protein–protein interaction. Several cysteine residues in these repeats were identified as targets for covalent modification by isothiocyanates, cinnamaldehyde, and acrolein known to be potent activators of TRPA1 [32]. Other activators of TRPA1 are histamine, prostaglandins, NGF, and bradykinin as important mediators of inflammation and pain after toxic injury [33]. Therefore, TRPA1 may mediate airway inflammation [34]. The channel was originally cloned in cultured fibroblasts [35] but also identified in cell lines of human lung fibroblasts (CCD19-Lu) and pulmonary epithelial cells (A549) [36]. The main in vivo expression, however, was found in neuronal tissues like spinal dorsal root ganglia (DRG), trigeminal ganglia [37], and bronchopulmonary afferent neurons, where the channel is co-expressed with TRPV1 [38] and is involved in detecting cold and hot temperatures as well as acrolein in cigarette smoke [39,40]. Moreover, TRPA1 protein was identified by immunohistochemistry in a subset of nodose ganglion neurons projecting to the lung and airway in mice [41]. Robust increases in intracellular Ca^2+^ were induced by hypoxia (10, 13, 15% O_2_) and hyperoxia (100% O_2_) in a subset of these neurons, which were also sensitive to capsaicin a specific activator of TRPV1 channels [42]. Thus, nodose neurons from TRPA1-deficient mice were largely unresponsive to hypoxia and hyperoxia [42] and TRPA1 was activated by both hypoxia and hyperoxia in vitro. While hypoxia induces dihydroxylation of the proline residue 349 in an aminoterminal Ankyrin repeat, hyperoxia increases crosslinking of two carboxy-terminal cysteines (Cys633 and Cys856) [42]. Moreover, ozone (O_3_), hypochlorite, and H_2_O_2_ as oxidizing agents activate TRPA1 as airborne irritants (reviewed in [33]). TRPA1 expression was also detected in non-neuronal tissues like lung epithelium by RT-PCR and immunohistochemistry [43] (see Table 5). However, as evidence for in vivo function of TRPA1 in non-neuronal lung tissues was still missing, we compared hyperoxia-induced increases in intracellular Ca^2+^ in TRPA1-/- and WT cells from tracheae, bronchi, and alveoli. While in all cells hyperoxia induced a rapid increase in intracellular Ca^2+^, no significant differences were detected in TRPA1-deficient cells compared to WT cells [44]. Along these lines, mice kept in a hyperoxic environment developed alveolar hyperplasia irrespective of their TRPA1 expression [44]. Although non-neuronal activation of TRPA1 was reported in airway inflammation [41], other TRPA1 functions in non-neuronal tissues remain elusive. TRPA1 is activated by a plethora of natural and artificial compounds [25], which cannot all be listed in a clear, structured table. Most interestingly, a lot of licensed drugs like acetaminophen [45], apomorphine [46], cannabinoids [45], chlorpromazine [47], oxaliplatin [48], chloroquine [49], etodolac [50], desflurane [51], and auranofin [52] activate TRPA1 and may be easily “repurposed/repositioned”, if TRPA1 activation is part of a therapeutic option. TRPA1 inhibitors are listed in Table 2. While compounds like HC-030031 [53], Chembridge (CB)-5861528 [54], and AP18 [55] are effective in a µmolar range, A967079 reaches IC_50_s of 67 and 289 nM on human and rat TRPA1, respectively [56]. AZ465 blocks only human, but not mouse TRPA1 [57] and GRC17536 [58] is the most promising compound to date, which is in a phase II clinical trial as a therapeutic option for chronic refractory cough (reviewed in [4,59]).

**TRPV1**, as the first cloned member of the vanilloid family of TRP channels, was identified in sensory neurons, which were activated by capsaicin the pungent ingredient in hot chilli peppers [61]. Capsaicin binding sites are located in the transmembrane region S3 and the loop region between S2 and S3 [62]. TRPV1 is expressed in 50% of small to medium size somatic and visceral neurons in dorsal root trigeminal and vagal ganglia [61,62]. Its expression is dependent on the production of nerve growth factor (NGF) or brain-derived neurotrophic factor (BDNF) as trophic signals [63,64]. Levels of both NGF and artemin, a member of the GDNF family, are increased during inflammation and up-regulated TRPV1 expression will contribute to thermal hypersensitivity [65]. Thus, TRPV1 contributes to heat (>42 °C) [66] and pain sensation [67]. A complete lack of the acute withdrawal response to noxious heat, however, was only observed in triple TRPV1-/-TRPM3-/-TRPA1-/- deficient mice [68]. Along these lines, heterotetrameric functional channels composed of TRPV1 and TRPA1 were identified and characterized [69].

Next to its prominent expression in neuronal cells, TRPV1 was also identified in arterial smooth muscle cells, where it may control blood flow [70,71], human bronchial fibroblasts [72], and lung epithelial cells [73,74] (see Table 5). In the latter tissue, recent evidence indicate that TRPV1 channels are good candidates for the detection of inhaled toxicants and their antagonists may protect from toxic lung injury and pain related to chronic inflammation (reviewed in [3]). Proposed roles of TRPV1 in inflammation of the lung during the development of asthma and chronic obstructive lung disease (COPD) are discussed below. Therefore, TRPV1 is the number one TRP-target for developing new drugs by numerous companies (reviewed in [25,75]). Historically, capsicum plasters containing capsaicin from chilli peppers were sold for more than 100 years against back pain without revealing the molecular basis of their action. Now, we know that TRPV1 channels are the target of capsaicin and are rapidly desensitized by complex cellular signaling events [76]. Along these lines, capsaicin patches are already provided for patients with postherpetic neuralgia (reviewed in [25]) and intranasal application of capsaicin provided beneficial effects to patients with vasomotor rhinitis, but was not well tolerated [77]. Thus, numerous TRPV1 inhibitors [25] were also developed and some selective compounds are listed in Table 3. Resolvin D2 also equally effects TRPA1 channels [78], while BCTC [79] and agatoxin AG489 [80] were not further tested for their selectivity. More selective is ASBT102, which is not effectively inhibiting TRPV4 and TRPA1 [81]. Pretreatment with SB-7054978 was effective in reducing capsaicin-induced secondary hyperalgesia [82], but a randomized, double blind, placebo-controlled cross-over trial on cough sensitivity to capsaicin was not successful [83]. The compound JNJ-17203212 was inhibiting osteoarthritis pain in rat [84] and bone cancer pain in a mouse model [85]. Very promising compounds like AMG517 from Amgen [86] and AZD1386 [87], which were in clinical trials for reducing pain after tooth extraction, increase body temperature. Therefore, the therapeutic value of TRPV1 antagonists are still under debate. For up-to-date information, please refer to Lee et al. [88]. Two phase II trials (NCT02233686 and NCT02233699) with the drug xention-D0501 ended in 2015, but no results were posted. Very recently, JNJ-39439335 (Mavatrep) was analyzed in a phase I study in healthy Japanese and Caucasian men as prospective pain reliever [89,90]. In contrast to other TRPV1 antagonists, the most common side effects thermohypoesthesia as well as feeling cold or hot were well tolerated by the volunteers [89]. 

The fourth member of the TRPV family, **TRPV4**, was identified in 2000 [93,94,95] and originally described as an osmo-sensitive channel [94], but is also sensitive to heat (<24 °C) [96], shear stress [97], and various chemical stimuli (see below). In human patients several mutations and single nucleotide polymorphisms (SNPs) in the TRPV4 gene are closely linked to different skeletal dysplasias, neuropathies, hyponatremia, as well as chronic obstructive pulmonary disease (COPD) (reviewed in [98] and see below). Like TRPV1, TRPV4 is expressed in the central nervous system and the brain, not only in neurons, but also in glial cells and astrocytes [98]. The channel senses intravesicular pressure in the bladder and is involved in regulation detrusor function (reviewed in [99]) and was identified in keratinocytes [100]. TRPV4 is also expressed in endothelial cells (reviewed in [101]), where it activates IK(Ca) channels for vasodilatation and blood flow autoregulation [102]. Next to bronchial epithelial cells [103], the channel is also functional in airway smooth muscle cells [104] and contributes to the ciliary beat frequency regulation in tracheal epithelial cells [105]. Serotonin-induced pulmonary vasoconstriction and the enhanced vascular reactivity in chronic hypoxic pulmonary vasoconstriction is dependent on TRPV4 [106]. TRPV4 activation reduces the alveolar barrier [107] and activates macrophages [108] contributing to acute lung injury. 

Along these lines, several reports confirm a role of TRPV4 in the disruption of the epithelial barrier and the formation of lung edema (reviewed in [109]). In other tissues however, the channel also contributes to the formation of the cellular barrier e.g. in skin [110], the urogenital tract [111] and the corneal epithelium [112]. Therefore, the application of TRPV4 antagonists may induce multiple harmful side effects. A selection of compounds [25] activating or inhibiting TRPV4 are listed in Table 4. The 4α phorbol esters [113] and epoxyeicosatrienoic acids (EET) [114] work as endogenous mediators of TRPV4. Bisandrographolide A [115] is a selective TRPV4 activators which does only weakly modulate other members of the vanilloid family like TRPV1, TRPV2, and TRPV3. GSK1016790A, the only in vivo tested so-called “superagonist”, causes a lethal drop in blood pressure in mice, cats, and dogs [116], while intravesical instillation caused bladder overactivity in mice [117]. Both effects, however, were only observed in wild-type, but not in TRPV4-deficient mice [116,117], emphasizing the important roles of the channel in endothelium-dependent vasorelaxation and bladder function. RN-1734 [118] and GSK205 [119] are moderate TRPV4 inhibitors blocking in the µM range, which were tested only in vitro. In vivo application of HC-067047 improved bladder function in mice and rats with cystitis induced by the application of the cytostatic compound cyclophosphamide [120]. GSK2193874 was effective in preventing pulmonary edema due to acute and chronic heart failure in mice [121]. This inhibitory effect on edema formation was reproduced in a human disease model-on-a-chip [122]. The probably most promising compound GSK2798745 was analyzed in phase I and phase II studies in patients with congestive heart failure and initial results are posted (NCT02119260 and NCT02497937 see https://clinicaltrials.gov/). 

## 3. Non-Neuronal TRPs as Potential Drug Targets in Lung Disease

**TRP** channels are expressed in many cells of the lung (see Figure 1) as outlined above and summarized in Table 5. In the upper airways and bronchi ciliated epithelial cells together with mucus producing goblet cells remove foreign particles. Airway smooth muscle cells (SMC) are able to constrict or relax the airways to meet the demand of oxygen in the body. The smaller bronchioles end in thousands of small air sacs called alveoli composed of alveolar type 1 (AT1) and alveolar type 2 (AT2) cells. The latter produce surfactant protein-C (SP-C) to facilitate gas exchange (O_2_, oxygen; CO_2_, carbon dioxide) through the alveolar capillary membrane with erythrocytes in the accompanying blood vessels. Alveolar macrophages (MP) clean the alveolus by phagocytosis. While the alveolar capillary membrane consists only of the alveolar epithelium, an interstitium (not depicted) and endothelial cells, precapillary, small and large pulmonary arteries are contractile by smooth muscle cells (e.g., precapillary arterial smooth muscle cells (PASMC). TRP channels do not seem to be essential for normal function of the lungs and airways, as TRP-deficient mice do not show any obvious pulmonary defects. Only for TRPV4-/- mice was an altered beat frequency of tracheal epithelial cells observed [105]. However, these proteins, as so-called “alarm channels”, are important for the compensation of pathophysiological conditions and may induce chronic diseases, like pain and decreased cell barrier function induced by over-active inflammation processes, if their activity is not strictly regulated. In these disease states, selective modulators of TRP channels are attractive new therapeutic options, which may induce only minor side effects, because normal cell function is not compromised. I will briefly introduce these pathological alterations in lung functions and summarize the involvement of pulmonary TRP channels in the progress of the disease in the following sections of this chapter.

### 3.1. Cystic Fibrosis (CF)

**Figure 2:**
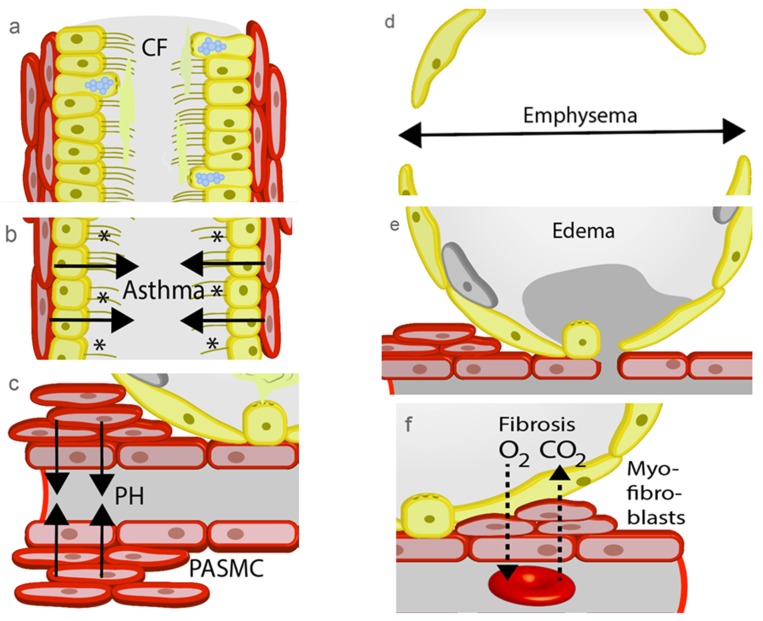
Pathophysiological changes in the lung. (**a**) Increasing viscosity of the mucus during the development of cystic fibrosis (CF) disables removal of foreign particles. (**b**) Contractions of the airways by allergens (*) occur during an asthma attack and prevents gas exchange. (**c**) Precapillary arterial smooth muscle cell (PASMC) contract to increase pulmonary blood pressure as an initial step in the development of pulmonary hypertension (PH). (**d**) A hallmark of chronic obstructive pulmonary disease (COPD) is the loss of alveolar septae during the development of emphysema. (**e**) Increased hydrostatic pressure by PH or damage of the alveolar capillary membrane induce lung edema. (**f**) Repair processes by myofibroblasts block gas exchange in the alveolar capillary membrane of patients with lung fibrosis.

CF patients carry mutations in the gene for the cystic fibrosis transmembrane conductance regulator (CFTR), an ion channel managing the passage of chloride and bicarbonate ions across the apical membrane of epithelial cells. Identified mutations in CF result in improper translation, processing and translocation of the CFTR protein to the plasma membrane as well as impaired conductance and regulation of the ion channel (reviewed in [129]). All mutated proteins, including the most common F508del CFTR mutation, induce production of high quantities of abnormal mucus of high viscosity (see Figure 2a), which is not suitable for the removal of particles as well as bacteria and viruses from the airways [129]. CF patients are suffering from compromised respiratory function, chronic infection in particular by *Pseudomonas aeruginosa*, and inflammation seriously damaging lung tissues [130]. 

The **TRPA1** protein was identified in bronchial epithelial cells and inhibition of its expression results in reduction of the release of several cytokines from these cells (IL-8, IL-1β, TNFα) in response to exposure to *P. aeruginosa* [131]. These data point to an important function of TRPA1 in airway inflammation during CF pathogenesis.

Most interestingly, CFTR down-regulates **TRPC6**-mediated Ca^2+^ influx, while TRPC6 up-regulates CFTR-mediated Cl^-^ transport, and both proteins physically interact with each other [132]. Therefore, TRPC6-mediated Ca^2+^ influx was increased in CF versus non-CF human epithelial cells, because functional coupling of CFTR and TRPC6 is lost. This mechanism called reciprocal coupling has also been observed in freshly isolated ciliated epithelial cells [132], which regulate mucus viscosity by CFTR activity. While the correction of transportation and function of the CFTR protein in epithelial cells was the main aim in the treatment of CF in the last years, a new paradigm also points to other possible targets. Dysregulated innate immunity [133] especially in neutrophils [134] and macrophages [135] is also responsible for defects in bacterial clearance observed in CF patients. Both cell types express TRPC6 proteins (see Table 5) as well as CFTR ion channels. Phagocytic capacity as well as fusion of phagosomes with lysosomes are not affected by mutated CFTR proteins, but bacteria are not destroyed and are even able to multiply in CF macrophages [136,137]. Moreover, CF neutrophils are unable to kill invading bacteria [138]. Most interestingly, a drug called roscovitine, which is tested in clinical studies as therapeutic option for a plethora of diseases, acts as a partial corrector of the F508del CFTR protein [139] and recruits TRPC6 translocation to phagosomal membranes, which is able to restore microbicidal function in alveolar macrophages compromised by CF [140]. Other functions of this drug on neutrophils, epithelial cells, eosinophils, and lymphocytes may also be beneficial to restore activity in CF affected cells (reviewed in [141]). 

**TRPV4** is important for the regulatory volume decrease (RVD) in airway epithelia, which is absent in CF airways, but could be recovered by 4α-phorbolesters as TRPV4 activators [142] (see Table 4). Specific inhibitors of these TRP channels need to be evaluated in clinical studies in the future.

### 3.2. Asthma

Asthma is a chronic inflammatory disease of the upper airways induced by repeated exposure to specific allergens, which results in activation of epithelial cells and acute bronchoconstriction (see Figure 2b). Differentiated T-helper 2 (Th2), mast cells, and eosinophils invade lung tissues and up-regulation of mediators of inflammation like IL-4, IL-5, IL-13 eotaxin (CCL11) and eicosanoids is observed. Patient suffer from symptoms like cough, dyspnea, wheezing, and chest tightness [143]. A longer-lasting response is also triggered by isocyanate [144], exercise, and cold air, releasing tachykinins and calcitonin gene-related peptide to promote neurogenic inflammation. Asthma patients inhale rapid and long-acting β_2_-adrenoceptor agonists for bronchodilation and glucocorticoids to inhibit chronic inflammation [145]. These current treatment options offer only symptomatic relief to the majority, but not all patients [146]. There are, however, no therapeutic options to date that are able to prevent or cure asthma [145]. 

Airway inflammation and hyper-reactivity can be mimicked in an established ovalbumine-induced mouse model. **TRPA1**-deficient (TRPA1^-/-^) mice or wild-type (WT) mice treated with the TRPA1 inhibitor HC-030031 (see Table 2) showed significant lower allergen-induced leucocyte infiltration, cytokine, and mucus production as well as airway hyper-reactivity [40]. The same was true in a mouse model of hypochlorite (ClO^−^) exposure followed by ovalbumin treatment. Here, airway hyper-responsiveness was induced without bronchial inflammation and was again significant reduced in TRPA1^-/-^ mice [147]. In both cases, however, global TRPA1-deficient mice were used and neuronal expressed TRPA1 channels were most probably responsible for disease symptoms. The role of TRPA1 in epithelial cells activated by e.g., isocyanates remains therefore elusive. 

In **TRPC6**-deficient mice, ovalbumine sensitization resulted in reduced allergic responses as evidenced by a decrease in airway eosinophilia and blood IgE levels, as well as decreased levels of Th2 cytokines (IL-5, IL-13) in the bronchoalveolar lavage [123]. Surprisingly, TRPC6^-/-^ mice exposed increased methacholin-induced bronchoconstriction due to compensatory up-regulation of related but constitutively active TRPC3 channels [123,148]. Along these lines, two recent reports were able to inhibit allergen-induced asthma by lentiviral knock-down or inhibition of TRPC3 [149,150].

**TRPV1** is significantly higher expressed in bronchial epithelia of asthma patients than in healthy volunteers [124] and a loss of function single nucleotide polymorphism (SNP) changing the amino acid isoleucine to valine (I585V) lowers the risk of active childhood asthma [151]. Contradictory data were produced with TRPV1 modulators. The use of two TRPV1 inhibitors (SB-705498 see Table 3 and PF-04065463) inhibited ovalbumin-induced airway hyper-responsiveness to histamine in guinea pig [91], while another blocker (SB366791) failed to reduce eosinophil infiltration in a mouse model of allergic asthma [152]. It is again, however, not clear, in which tissue (neuronal or non-neuronal) TRPV1 is mediating allergic responses to asthma.

In a house dust mite model, which is more relevant to the human situation, **TRPV4**-/- mice were protected from airway remodeling and fibroblasts isolated from asthmatic human patients exposed increased TRPV4 activity compared to cells from healthy donors. Therefore, TRPA1, TRPV1, and TRPV4 are most promising pharmacological targets for new asthma therapeutics and already effective modulators are ready for use in clinical trials.

### 3.3. Pulmonary Hypertension (PH)

PH by remodeling of the pulmonary vessels leads to a progressive increase in pulmonary vascular resistance, due to proliferation and constriction of smooth muscle cells in the precapillary small arteries (see Figure 2c), which are responsible for blood pressure regulation in the lung. PH can be induced by left heart failure, chronic lung disease, hypoxia, thromboembolic events, or other unknown multifactorial mechanisms (reviewed in [153]). Hitherto known therapeutic options include endothelin receptor antagonists, phosphodiesterase 5 inhibitors, guanylyl cyclase stimulators, prostaglandin receptor agonist, and prostaglandin analogues [154].

**TRPC6** is essential for the acute phase of hypoxia-induced pulmonary vasoconstriction (HPV), which is absent in TRPC6-/- mice [18] and can be reduced by a TRPC6 selective larixylmethyl-derivate (see Table 1 and [29]). Data for remodeling processes induced by chronic hypoxia (10% O_2_ for ≥3 weeks), however, are contradictory. We have demonstrated that TRPC6-deficient mice display chronic hypoxia-induced PH indistinguishable from those in wild-type (WT) mice [18]. In TRPC1/6-double-deficient mice, PH was decreased after exposure to chronic hypoxia and muscularization of lung microvessels was inhibited compared to WT mice [155], indicating that both TRPC channels may be responsible for the development of PH. In two other reports, notch-induced up-regulation of TRPC6 [156] and CFTR-mediated translocation of TRPC6 to caveolae [157] were responsible for chronic PH. While strain and sex differences may be responsible for the different results, all these observations in mouse models clearly demand more studies in human patients. In fact, mRNA and protein expression of TRPC6 in lung tissues and PASMCs from patients with idiopathic pulmonary arterial hypertension (IPAH), which develop the disease due to unknown reasons, is much higher than in those from normotensive patients [158]. Furthermore, inhibition of TRPC6 gene expression by small interfering RNA (siRNA) significantly diminished proliferation of PASMCs from IPAH patients [158]. Moreover, TRPC6 regulatory regions of 268 patients with IPAH revealed three SNPs. One of them (−254(C > G)) increased basal TRPC6 gene promoter activity and introduces a new binding site for the inflammatory transcription factor κB (NFκB) [159] probably mediating inflammatory mechanisms, which are also observed during the development of PH [160]. 

Similar results like for TRPC6 were demonstrated for **TRPV4**, which is also responsible for acute and chronic hypoxic vasoconstriction [161,162] and might form functional heteromeric channels with TRPC6, as both co-immunoprecipitate in PASMC lysates [162]. 

Thus, specific TRPC6 and TRPV4 inhibitors (see Table 1 and Table 4) might be successful in the therapy of PH in the future.

### 3.4. Chronic Obstructive Pulmonary Disease (COPD)

Chronic bronchitis, bronchiolitis, and emphysema are summarized by the term COPD. While the first two pathologies result in cough, fever, and dyspnea, the development of emphysema is characterized by an enlargement of alveoli (see Figure 2d), causing shortness of breath [3,163]. The vast majority of COPD patients are smokers [164], but exposure to wood smoke is also an important initiator of the disease [165]. Activation of lung resident immune cells, accumulation of inflammatory cells, e.g., neutrophils, and secretion of IL-1β and IL-8 are typical symptoms of COPD. In addition to drugs used for the treatment of asthma, parasympatholytics like ipratropium bromide serve as therapeutics, but are no causal intervention and are far from curing the disease [3].

As outlined above, acrolein from cigarette smoke activates **TRPA1** and an acrolein-induced increase in IL-8 secretion is significantly blocked by the TRPA1 antagonists AP18 (see Table 2) [60]. 

Airway inflammation in COPD patients is closely related to the invasion of neutrophils, because neutrophil counts were increased in the sputum of COPD patients and correlate to their disease state [166]. **TRPC6** is expressed in neutrophils, is important for their migration [23,24] and may contribute to the progress of the disease. Moreover, TRPC6 mRNA was significantly up-regulated in lung macrophages of smokers, while TRPC3 and TRPC7 mRNA expression was similar in comparison to non-smokers [22]. 

Along the same line, the **TRPV1** activator capsaicin raised IL-8 secretion dose dependently from airway epithelial cells [124] and induced increased cough response in COPD patients compared to healthy volunteers by activating neuronal TRPV1 [167]. Strikingly, TRPV1 and **TRPV4** mRNA levels were increased in samples from patients with COPD and cigarette-exposure-induced ATP release from primary bronchial epithelial cells was attenuated by blockers of both channels (JNJ1703212, HC087047 see Table 3 and Table 4, [92]). Furthermore, increased levels of TRPV4 transcripts by gene polymorphisms are associated with COPD [168] and TRPV4 is highly expressed in alveolar macrophages (see Table 5). In summary, data for an involvement of TRP channels in the development of COPD are promising and should justify further studies in patients.

### 3.5. Lung Edema

Flooding of the alveoli due to lung edema (see Figure 2e) hampers gas exchange and may induce life threatening arterial hypoxemia. Endothelial dysfunction in the pulmonary vessels by toxic agents e.g., cytostatic drugs and smoke adducts circulating in the blood is one important reason for edema formation. Increased hydrostatic pressure by left heart failure leads to higher ventricular pressure transmitting back to the pulmonary circulation and may also increase endothelial permeability as well as trigger edema formation [3]. During pulmonary inflammation in patients with acute respiratory distress syndrome (ARDS) mediators like platelet-activating factor (PAF) [169] are also able to decrease endothelial barrier function in between minutes. In a fourth mechanism ischemia increases endothelial permeability initiating edema in isolated lungs intended for transplantation. Subsequently, invading immune cells are responsible for inflammation destroying urgently needed lung transplants [170]. 

**TRPC6** and TRPV4 are expressed in endothelial cells (see Table 5) and there are numerous reports presenting evidence for their involvement in lung edema formation. We were able to demonstrate an essential role of TRPC6 in lung ischemia-reperfusion-induced edema (LIRE) formation and developed a novel mechanistic model [19] (see above). Most interestingly, a larixylderivate as selective TRPC6 blocker (see Table 1) was able to inhibit LIRE [30] a significant cause of primary graft failure in lung transplantation technology [170]. Translocation of TRPC6 to caveolae enriched with ceramide, an important product of the PAF signaling pathway, was found to be enhanced in pulmonary endothelial cells after application of PAF [171]. The resulting Ca^2+^ influx through TRPC6 can at least partly explain increases in endothelial permeability. Lipopolysaccharide (LPS) as an endotoxin produced by invading bacteria in septic patients is also able to activate TRPC6 and increase endothelial permeability in a Toll-like receptor 4 (TLR4)-dependent manner [172]. LPS binding to TLR4 induces DAG production by a still unknown mechanism, which activates TRPC6 [172]. Therefore, TRPC6 inhibitors may also help to prevent acute lung injury in septic patients.

Along the same lines, **TRPV4**, a key regulator of lung endothelial barrier function (reviewed in [101,109]), is directly activated by LPS independently of and faster than the TLR4 pathway [173]. TRPV4-deficency reduces hydrostatic lung edema formation and capillary leakage [174,175], which can be explained by stretch induced activation of TRPV4 resulting in increased endothelial Ca^2+^ [176] and activation of endothelial NO production [177]. Most interestingly, the TRPV4 blocker GSK2193874 (see Table 4) was effective in inhibiting lung edema by high pulmonary venous pressure as well as in a myocardial infarction mouse model [121]. Vice versa, activation of TRPV4 by 4α-phorbol esters (see Table 4) initiates lung edema [178]. Acid aspiration by gastroesophageal reflux results in acute lung injury with infiltration of macrophages and neutrophils. Protection from these symptoms was achieved in TRPV4-/- mice as well as in wild-type mice after application of TRPV4 blockers (GSK2220961, GSK2337429A) in a therapeutic approach 30 min after exposure, thus assembling the clinical situation [125]. GSK2193874 (see Table 4), however, needed to be applied before injury and showed only a prophylactic effect [175]. Proposed molecular mechanisms of edema formation by TRPV4 include Ca^2+^-induced activation of myosin light chain kinase (MLCK) in endothelial cell (reviewed in [101]) as well as opening of Ca^2+^-activated K^+^ channels (KCa3.1) in epithelial cells inducing hyperpolarization [179]. The latter effect may provide the driving force for Cl^−^ through Ca^2+^ activated Cl^−^ (CaCC) as well as CFTR channels causing extracellular liquid accumulation (reviewed in [180]). Therefore, TRPC6 and TRPV4 are attractive targets for developing new therapeutic options for patients with lung edema. 

### 3.6. Lung Fibrosis

An impaired repair process after chronic lung damage by toxicants (e.g., the cytostatic drug bleomycin) is able to induce lung fibrosis, which is characterized by an accumulation of fibroblasts differentiating to myofibroblasts in response to secreted profibrotic transforming growth factor β (TGF-β1) [181]. Myofibroblasts express α-smooth muscle actin as well as secret large amounts of extracellular matrix, e.g., collagens and accumulate in fibroproliferative foci, which inhibit gas exchange (Figure 2f). Despite recent progress with the approval of the drugs pirfenidone and nintedanib for the treatment pulmonary fibrosis, the only causative treatment is still lung transplantation. 

Deficiencies of TRPC6 [21] and TRPV4 [128] independently protected lungs in a mouse model of bleomycin-induced lung fibrosis. While **TRPC6** is not expressed in quiescent fibroblasts but is up-regulated by TGF-β1 and is involved in myofibroblasts differentiation [21], **TRPV4** is constitutively expressed in lung fibroblasts and may be engaged by mechanical stress [128]. Although many different cell types are involved in the development of lung fibrosis [181], both reports exclusively analyzed fibroblasts–myofibroblast-transdifferentiation and employed globally gene-deficient mice. Only tissue specific TRP-deficiency will answer the question which cells apart from fibroblasts are responsible for the development of lung fibrosis due to TRPC6 or TRPV4 function. 

## 4. Conclusions

As outlined above, TRP channels do not contribute to normal lung function, but are involved in repair processes (e.g., increases in endothelial permeability, inflammation, myofibroblast differentiation) which might become pathologic. Therefore, they are ideally suited as pharmacological targets to develop new therapeutic options with less side effects. The most compounds already tested in clinical phases exist for TRPV1 as new drugs for pain relief, followed by modulators for TRPV4 efficient against lung edema. Only a few drugs exist for regulating TRPC6 activity and only one of them was successfully blocking lung edema in vitro. The same is true for TRPA1 with one drug being successful in the reduction of airway inflammation in a mouse model of asthma. In summary, translational research in patients and human tissues still have to validate initial data obtained in animal experiments and phase I and II clinical studies. In case of positive results, specific modulators of these channels may serve as new therapeutic options acting after resorption from the vasculature or from the airways after inhalation.

## Figures and Tables

**Table 1:** Compounds regulating TRPC6 activity and prospective therapeutic options.

Drug	TRPC6	TRPC3	TRPA1	TRPV1	Therapeutic Opt.	Ref.
Flufenamic ac.	+	-	+	-	?	[25]
Aniline-thiazole	-	-	?	?	?	[26]
GSK comp.	-	-	?	?	Heart hypertrophy	[27]
Larixyl deriv.	-	/	/	/	Lung edema	[29,30]
SAR7334	-	-	?	?	?	[28]
BTDM	-	-	?	?	?	[31]

+, activating; -, inhibiting; ?, not tested; /, very low activity, ac., acid, GSK comp., GSK compounds GSK2332255B and GSK2833503A; Ref. reference.

**Table 2:** Compounds inhibiting TRPA1 activity and prospective therapeutic options.

Drug	TRPA1	TRPV1	TRPV4	Therapeutic Opt.	Ref.
HC-030031	-	?	?	Asthma	[40,53]
CB-5861528	-	?	?	?	[54]
AP18	-	/	/	Toxic lung inj.	[55,60]
A967079	-	/	/	?	[56]
AZ465	- ^1^	?	?	Cough	[57]
GRC17536	-	?	?	?	[58]

^1^ human TRPA1; -, inhibiting; ?, not tested; /, very low activity; opt. options; Ref., reference.

**Table 3:** Selected compounds inhibiting TRPV1 activity and prospective therapeutic options.

Drug	TRPV1	TRPV4	TRPA1	Therapeutic Opt.	Ref.
Resolvin D2	-	?	-	-	[78]
BCTC	-	?	?	-	[79]
Agatoxin AG489	-	?	?	-	[80]
ABT102	-	/	/	-	[81]
AMG517	-	?	?	Tooth pain	[86]
SB-7054978	-	?	?	Hyperalgesia	[82]
“				Asthma	[91]
AZD-1386	-	?	?	Tooth pain	[87]
JNJ-17203212	-	?	?	Osteoarthritis pain	[84]
“				Bone cancer pain	[77]
“				COPD	[92]
JNJ-39439335	-	?	?	various pain cond.	[89]

-, inhibiting; ?, not tested; /, very low activity; opt. options; Ref., reference.

**Table 4:** Selected compounds activating or inhibiting TRPV4.

Drug	TRPV4	TRPV1	TRPV2/3	TRPM8	Therapeutic Opt.	Ref.
4α-phorbolesters	+	/	/	?	-	[113]
EETs	+	?	?	?	-	[114]
Bisandrograph.	+	/	/	?	-	[115]
GSK1016790A	+	/	?	?	Blood pressure ↓	[109]
RN1734	-	/	/	/	-	[118]
GSK205	-	?	?	?	-	[119]
HC-067047	-	/	/	/	Cystitis	[120]
“					COPD	[92]
GSK2193874	-	?	?	?	Pulm. edema	[121]

+, activating; -, inhibiting; ?, not tested; /, very low activity; ↓, reduction, opt., options, Ref., reference.

**Table 5:** TRP expression patterns in lung cells.

Lung Cell	TRPA1	TRPC6	TRPV1	TRPV4
Bronchial epi.	−/+[44] ^1^, [43] ^3^	+[123] ^2^	+[124] ^3^	+[92] ^2^
Airway SMC	−/+[44] ^1^	+[123] ^2^	+[70] ^3^	+[104] ^2^
AT1 cells	−/+[44] ^1^	?	?	?
AT2 cells	−/+[44] ^4^	?	?	?
Alveolar MP	?	+[22] ^4^	?	+[125] ^5^
Endothelium	−/+[44] ^1^	+[19] ^4^	+[126] ^2^	+[126] ^2^
PASMC	−/+[44] ^1^	+[18] ^2^	+[127] ^4^	+[127] ^2^
Neutrophils	?	+[23] ^3^	?	+[23] ^2^
Fibroblasts	−/+[44] ^1^	−/+[21] ^2^	?	+[128] ^4^
Myofibroblasts	?	+[21] ^4^	?	+[128] ^4^

−/+, very low expression, +, expression, ++, high expression, ?, not tested. References in brackets []. Detection by: ^1^ labeling of specific mRNA (nanostring® technology); ^2^ amplification of specific mRNA by quantitative reverse transcription (qRT)-PCR; ^3^ imunohistochemistry, ^4^ labeling protein by specific antibodies in a Western Blot, ^5^ functional assays.
